# Clinical biomarkers of pulmonary carcinoid tumors in never smokers via profiling miRNA and target mRNA

**DOI:** 10.1186/2045-3701-4-35

**Published:** 2014-07-09

**Authors:** Bo Deng, Julian Molina, Marie C Aubry, Zhifu Sun, Liang Wang, Bruce W Eckloff, George Vasmatzis, Ming You, Eric D Wieben, Jin Jen, Dennis A Wigle, Ping Yang

**Affiliations:** 1 Department of Health Sciences Research, Mayo Clinic College of Medicine, Rochester, Minnesota, USA; 2 Thoracic Surgery Department, Institute of Surgery Research, Daping Hospital, Third Military Medical University, Chongqing, People’s Republic of China; 3 Department of Oncology, Division of Medical Oncology, Mayo Clinic College of Medicine, Rochester, Minnesota, USA; 4 Department of Laboratory Medicine and Pathology, Mayo Clinic College of Medicine, Rochester, Minnesota, USA; 5 Department of Pathology, Medical College of Wisconsin Cancer Center, Milwaukee, Wisconsin, USA; 6 Medical Genome Facility, Mayo Clinic College of Medicine, Rochester, Minnesota, USA; 7 Department of Molecular Medicine, Mayo Clinic College of Medicine, Rochester, Minnesota, USA; 8 Department of Cancer Center and Toxicology, Medical College of Wisconsin, Milwaukee, Wisconsin, USA; 9 Department of Biochemistry and Molecular Biology, Mayo Clinic College of Medicine, Rochester, Minnesota, USA; 10 Department of Surgery, Division of General Thoracic Surgery, Mayo Clinic College of Medicine, Rochester, Minnesota, USA

**Keywords:** miRNA, mRNA, Carcinoid, Survival

## Abstract

**Background:**

miRNAs play key regulatory roles in cellular pathological processes. We aimed to identify clinically meaningful biomarkers in pulmonary carcinoid tumors (PCTs), a member of neuroendocrine neoplasms, via profiling miRNAs and mRNAs.

**Results:**

From the total of 1145 miRNAs, we obtained 16 and 17 miRNAs that showed positive and negative fold changes (FCs, tumors vs. normal tissues) in the top 1% differentially expressed miRNAs, respectively. We uncovered the target genes that were predicted by at least two prediction tools and overlapped by at least one-half of the top miRNAs, which yielded 44 genes (FC<-2) and 56 genes (FC>2), respectively. Higher expressions of *CREB5*, *PTPRB* and *COL4A3* predicted favorable disease free survival (Hazard ratio: 0.03, 0.19 and 0.36; P value: 0.03, 0.03 and 0.08). Additionally, 79 mutated genes have been found in nine PCTs where TP53 was the only repeated mutation.

**Conclusion:**

We identified that the expressions of three genes have clinical implications in PCTs. The biological functions of these biomarkers warrant further studies.

## Background

Pulmonary carcinoid tumors (PCTs) are a member of lung neuroendocrine neoplasm family. The incidence of PCTs is relatively low and comprises approximately 1-2% of lung neoplasms [1-3]. PCTs occur frequently in never-smokers, and its molecular etiology is still unclear. PCTs are subdivided into typical carcinoids (TCs) and atypical carcinoids (ACs), where TCs lack necrosis and have < 2 mitoses/2 mm^2^, and ACs have 2-10 mitoses/2 mm^2^ and/or necrosis [3]. Seventy to ninety percent of PCTs are identified as TCs and 10-30% as ACs [4]. The overall survival of cases undergoing radical resection are relatively satisfactory: 92-100% for TCs and 61-88% for ACs [3,5]. However, surgical resection is still the only effective treatment, and unresectable tumors are very difficult to treat due to their insensitivity for both radiation and chemotherapy. Additionally, recurrence or metastasis can occur even decades after primary resection [6].

miRNAs are genomically encoded small non-coding RNAs that regulate genetic information by controlling stability or translation of mRNAs. Thus far, the study on exploring miRNA biomarkers and their target genes for disease monitoring or as a treatment option is state-of-the-art, timely and highly promising [7,8]. We believe the profiling of miRNAs and screening of their target mRNAs are important to elucidate the tumorigenesis mechanisms of PCTs and indicate the potential directions of targeted therapy. In the study, we investigated the differential expression of miRNAs and target mRNAs. Additionally, we analyzed the clinical implications of the expression of these miRNAs and mRNAs in PCTs.

## Results

Fifty-one individuals who had never smoked tobacco products and developed PCTs between 1997 and 2008 were studied. All study protocols were approved by Mayo Clinic’s Institutional Review Board. The clinical and demographical features of these cases are shown in Table 1.

**Table 1:** The clinical and demographical characteristics of pulmonary carcinoid cases

	**Cases for DASL-miRNA (N=47)**	**Cases for DASL-mRNA (N=51)**	**Cases for RNA-seq & Exon-seq (N=9)**
**Age at diagnosis**			
N	47	51	9
Mean (SD)	62.2 (17.0)	63.0 (16.3)	57.4 (18.5)
**Gender**			
Female	31 (65.9%)	35 (68.6%)	6 (66.7%)
Male	16 (34.1)	16 (31.4%)	3 (33.3%)
**Cell type**			
Typical	44 (93.6%)	48(94.1%)	8 (88.9%)
Atypical	3 (6.4%)	3(5.9%)	1 (11.1%)
**Tumor stage**			
Stage I & II	36 (76.6%)	40 (78.4%)	9 (100%)
Stage III & IV	11 (23.4%)	11 (21.6%)	0 (0%)
**Sample set**			
FFPE	23 (48.9%)	26 (50.9%)	--
FF	24 (51.0%)	25 (49.0%)	9 (100%)
**Median follow**-**up period (year)**	6.4	6.4	7.2
**Events**			
Yes	5 (10.6%)	5 (9.8%)	0 (0.00%)
No	42 (89.4%)	46 (90.2%)	9 (100%)

### Sources of variation (SOV) and principal components analysis (PCA) of miRNAs expression

As indicated in the Additional file 1: Figures S1A and B, tumor status (tumor vs. normal) was the most effective factor with regard to SOV of miRNAs expression. Plate effects that were resulting from the split of the testing into manageable rounds, i.e., batch effects within each sample type, had the second highest significant F ratio. Hence, we used the plate effects as the covariance in the ANOVA model for differential analysis between tumor and normal.

As indicated in Additional file 1: Figures S1C and D, PCA indicates that miRNA expressions between tumors vs. normal tissues of formalin-fixed paraffin-embedded tissues (FFPE) were better separated than fresh-frozen (FF) samples.

### The profiling of miRNAs in the top 1% of fold changes (FCs)

We identified the median FC, Bonferroni P-value and False Discovery Rate (FDR)-Q-value for all of the 1145 miRNAs. We sorted miRNAs by FC in FFPE and FF sample sets, respectively. Thereafter, we selected the top 12 (12/1145, i.e., 1%) in FF and FFPE, respectively. Table 2 lists the 16 miRNAs that showed a positive FC. Among the 16 miRNAs, eight miRNAs are the top 12 in both of the sample sets, and the other eight miRNAs are the top 12 in either of the sample sets. Table 3 lists the top 17 miRNAs that showed a negative FC. Among the 17 miRNAs, seven miRNAs are the top 12 in both of the sample sets, and the other ten miRNAs are the top 12 in either of the sample sets.

**Table 2:** **Top differentially-expressed miRNAs with positive fold changes**

**miRNA**	**Bonferroni P-value (FF sample set)**	**FDR (Q-value) (FF sample set)**	**Fold change (FF sample set)**	**Bonferroni P-value (FFPE sample set)**	**FDR (Q-value) (FFPE sample set)**	**Fold change (FFPE sample set)**
miR-129*	**2.80E-09**	**4.49E-11**	*9.60123*	**5.14E-17**	**1.56E-18**	*14.0359*
miR-323-3p	**4.80E-13**	**9.24E-14**	*10.8586*	**1.88E-20**	**2.57E-21**	*12.0982*
miR-487b	**1.26E-09**	**3.03E-11**	5.73129	**7.55E-20**	**6.89E-21**	*11.3349*
miR-410	**8.52E-10**	**2.73E-11**	*7.64796*	**1.14E-23**	**3.13E-24**	*11.1012*
miR-369-3p	**4.16E-09**	**5.52E-11**	5.73725	**1.13E-14**	**1.93E-16**	*8.76275*
miR-376a*	**7.08E-11**	**4.54E-12**	*6.60479*	**2.16E-19**	**1.48E-20**	*8.21775*
miR-432	**1.88E-08**	**1.90E-10**	5.77297	**2.36E-16**	**5.38E-18**	*8.18243*
miR-129-3p	**6.07E-07**	**4.10E-09**	5.51091	**4.92E-12**	**5.61E-14**	*8.02623*
miR-409-3p	**3.84E-07**	**2.69E-09**	*6.53245*	**1.18E-13**	**1.70E-15**	*7.97883*
miR-494	**3.15E-08**	**2.88E-10**	*5.83220*	**5.39E-12**	**5.90E-14**	*7.91744*
miR-376a*:9-1	**2.64E-09**	**4.42E-11**	*7.65026*	**1.45E-11**	**1.42E-13**	*7.50156*
miR-136*	**1.22E-09**	**3.03E-11**	*6.24329*	**1.24E-16**	**3.08E-18**	*7.41737*
miR-370	**1.85E-09**	**3.94E-11**	*6.11260*	**4.18E-15**	**8.80E-17**	7.24492
miR-127-3p	**7.21E-10**	**2.52E-11**	*6.92131*	**3.33E-19**	**1.82E-20**	7.15394
miR-154	**7.86E-08**	**6.43E-10**	*6.29887*	**4.48E-18**	**1.75E-19**	6.79683
miR-376a	**1.64E-09**	**3.70E-11**	*7.54254*	**4.63E-10**	**3.53E-12**	6.19791

Note: We sorted miRNA by positive fold changes of FFPE and FF sample sets, respectively. We selected the top 12 in FF and FFPE of which the fold changes are denoted in italics, respectively. Eight miRNAs are the top 12 in both of the sample sets, and the other eight miRNAs are the top 12 in either of the sample sets. If Bonferroni P-value or FDR (Q-value) are <0.05 or <0.01, they are labeled in bold font, respectively. *FF*=Fresh Frozen tissues.

*FF*=Fresh Frozen tissues.

*FFPE*=Formalin-Fixed Paraffin-Embedded tissues.

*FDR*=False Discovery Rate.

**Table 3:** **Top differentially-expressed miRNAs with negative fold changes**

**miRNA**	**Bonferroni P-value (FF sample set)**	**FDR (Q-value) (FF sample set)**	**Fold change (FF sample set)**	**Bonferroni P-value (FFPE sample set)**	**FDR(Q-value) (FFPE sample set)**	**Fold change (FFPE sample set)**
miR-203	**7.65E-12**	**9.81E-13**	*−5.27985*	**2.53E-08**	**1.40E-10**	*−3.47194*
miR-224	**3.35E-09**	**4.77E-11**	*−3.98143*	**6.67E-07**	**3.26E-09**	*−3.02567*
miR-155	**4.18E-08**	**3.73E-10**	*−3.79316*	**1.98E-08**	**1.13E-10**	*−3.00440*
miR-302d	1	0.0280996	−2.07804	0.213123	**0.000384031**	*−2.89387*
miR-34b	**6.04E-05**	**2.53E-07**	*−3.97282*	0.0647553	**0.000135572**	*−2.84861*
miR-181b	**6.15E-11**	**4.54E-12**	−2.78165	**2.56E-10**	**2.06E-12**	*−2.84466*
miR-193a-5p	**6.09E-06**	**2.96E-08**	−2.47796	**2.56E-08**	**1.40E-10**	*−2.82774*
miR-34b*	0.202781	**0.0003304**	−2.35874	**0.0239414**	**5.51E-05**	*−2.68848*
miR-222	**6.14E-09**	**7.38E-11**	*−3.23244*	**0.0021736**	**6.33E-06**	*−2.66284*
miR-30a-3p	**5.59E-10**	**2.38E-11**	*−3.68236*	**0.00758472**	**2.00E-05**	*−2.65532*
miR-938	1	0.032323	−2.11394	0.558499	**0.000831352**	*−2.64146*
miR-218	**9.63E-07**	**6.17E-09**	*−3.60068*	**0.00014186**	**5.71E-07**	*−2.54212*
miR-511	**6.18E-10**	**2.38E-11**	*−3.62032*	**2.35E-05**	**1.01E-07**	−2.41325
miR-34c-3p	**2.70E-05**	**1.16E-07**	*−3.12580*	**0.0165783**	**3.88E-05**	−2.37615
miR-10a	**1.03E-06**	**6.37E-09**	*−3.11085*	0.306927	**0.000528712**	−2.07425
miR-146a	**5.81E-06**	**2.87E-08**	*−3.13742*	**0.0120827**	**2.95E-05**	−1.91491
miR-1	**3.78E-11**	**3.63E-12**	*−3.16869*	0.339905	**0.000564228**	−1.75322

Note: We sorted miRNA on negative fold changes of FFPE and FF sample sets, respectively. We selected the top 12 in FF and FFPE of which the fold changes are denoted in italics, respectively. Seven miRNAs are the top 12 in both of the sample sets, and the other ten miRNAs are the top 12 in either of the sample sets. If the Bonferroni P-value or FDR (Q-value) are <0.05 or <0.01, they are labeled in bold font, respectively. Six miRNAs met all the criteria, i.e., Bonferroni P-value<0.05, FDR (Q-value)<0.01, and top fold changes in both sample sets are indicated by the deep shaded rows.

*FF*=Fresh Frozen tissues.

*FFPE*=Formalin-Fixed Paraffin-Embedded tissues.

*FDR*=False Discovery Rate.

Table 2 indicates that FCs ranged from 10.86 to 5.51 and from 14.04 to 6.19 in FF and FFPE sample sets, respectively. Table 3 indicates FCs ranged from -5.28 to -2.08 and from -3.47 to -1.75 in FF and FFPE sample sets, respectively. The absolute value of positive FCs were remarkably greater compared to the absolute values of negative FCs, suggesting PCTs are prone to have inhibited tumor suppressor genes via high expression of miRNAs.

### The profiling of target mRNAs with FCs>2 or <-2

To screen the target mRNAs of the miRNAs as in the top 1% of FCs, we used prediction tools to predict the target mRNAs of the miRNAs listed in Tables 2 and 3, respectively. Thereafter, we uncovered the target genes which were predicted by at least 2 prediction tools and overlapped by at least one-half of the miRNAs in Table 2 (8 of 16 miRNAs) and Table 3 (9 of 17 miRNAs), respectively.

The expressions of 680 and 792 target genes, which were derived from the miRNAs in Tables 2 and 3, were finally confirmed in the DASL (cDNA-mediated Annealing Selection Extension and Ligation) assay, respectively. We listed all 44 genes with a median FC<-2 (expressions in tumors are at least two-fold lower than in normal tissues, Table 4) and 56 genes with a FC>2 (expressions in tumors are at least two-fold higher than in normal tissues, Table 5), respectively.

**Table 4:** **44 target genes with fold changes**<-**2 in either FF or FFPE**

**Gene**	**Bonferroni P-value (FF sample set)**	**FDR (Q-value) (FF sample set)**	**Fold change (FF sample set)**	**Bonferroni P-value (FFPE sample set)**	**FDR (Q-value) (FFPE sample set)**	**Fold change (FFPE sample set)**
*GLDN*	**3.17E-08**	**4.20E-10**	*−5.68982*	**0.000135**	**4.60E-06**	*−2.52804*
*CLIC5*	**1.10E-06**	**7.14E-09**	*−5.29096*	**0.000128**	**4.60E-06**	*−3.65321*
*GBP5*	**3.95E-07**	**3.49E-09**	*−4.70136*	0.270449	**0.002037**	*−3.34128*
*CPM*	**7.00E-11**	**8.53E-12**	*−4.22426*	**6.24E-06**	**3.19E-07**	*−2.34316*
*ATOH8*	**7.34E-09**	**1.40E-10**	*−3.94674*	0.057324	**0.000599**	*−3.35103*
*EXPH5*	**3.96E-09**	**8.90E-11**	*−3.62151*	0.339217	**0.002413**	*−2.18435*
*DKK2*	**1.03E-09**	**3.00E-11**	*−3.47820*	0.135328	**0.001155**	*−2.27702*
*CUGBP2*	**1.18E-07**	**1.23E-09**	*−3.42984*	**5.43E-07**	**4.64E-08**	*−2.62785*
*AQP4*	**0.000278**	**8.64E-07**	*−3.37647*	**4.22E-05**	**1.80E-06**	*−2.62410*
*COL4A3*	**2.88E-06**	**1.62E-08**	*−3.29984*	**0.003631**	**6.89E-05**	*−2.31972*
*LATS2*	**2.90E-08**	**4.04E-10**	*−3.26820*	0.084228	**0.000784**	*−2.67462*
*CXCL5*	**0.002516**	**6.07E-06**	*−3.19053*	1	0.028874	−1.51815
*BNC2*	**1.13E-06**	**7.16E-09**	*−2.95318*	**1.42E-05**	**6.62E-07**	−1.97542
*CDKN2B*	**8.98E-09**	**1.54E-10**	*−2.92826*	0.350879	**0.002462**	*−2.06729*
*HAS3*	**2.39E-06**	**1.37E-08**	*−2.89151*	1	0.041676	−1.52060
*HNMT*	**8.23E-10**	**2.67E-11**	*−2.69590*	**0.000877**	**1.95E-05**	−1.80218
*BACH2*	**7.75E-07**	**5.52E-09**	*−2.68793*	0.05984	**0.000613**	*−2.47454*
*NOTCH2*	**4.20E-06**	**2.19E-08**	*−2.68482*	1	0.011439	−1.98234
*PHACTR2*	**1.09E-05**	**4.92E-08**	*−2.66216*	1	0.011696	*−2.13119*
*AFF3*	**0.00117**	**3.08E-06**	*−2.64327*	0.330617	**0.002385**	*−2.19623*
*PTPRB*	**2.98E-07**	**2.86E-09**	*−2.54037*	**0.006134**	**0.000105**	−1.76936
*IRAK3*	**3.10E-06**	**1.68E-08**	*−2.50010*	0.06931	**0.000683**	−1.90606
*ARHGAP28*	**1.24E-09**	**3.23E-11**	*−2.49453*	**0.00221**	**4.53E-05**	*−2.07438*
*ARHGAP29*	**8.23E-08**	**9.24E-10**	*−2.46960*	0.462337	**0.002924**	*−2.11428*
*CCDC68*	**9.93E-07**	**6.74E-09**	*−2.46136*	0.252319	**0.00202**	−1.79536
*CENTD1*	**4.58E-05**	**1.74E-07**	*−2.45484*	**0.000286**	**7.99E-06**	*−2.37418*
*PDGFRA*	**8.30E-07**	**5.76E-09**	*−2.45213*	1	0.015458	−1.80164
*IGF1*	**0.000385**	**1.17E-06**	*−2.33199*	1	0.017787	−1.55397
*MAF*	**1.47E-08**	**2.38E-10**	*−2.32004*	1	0.022448	−1.74096
*CREB5*	**7.68E-09**	**1.40E-10**	*−2.29795*	**0.000166**	**5.31E-06**	−1.80701
*QKI*	**5.96E-10**	**2.32E-11**	*−2.26821*	**0.0408**	**0.000498**	*−2.11396*
*OBFC2A*	**5.84E-05**	**2.10E-07**	*−2.22977*	**0.000653**	**1.52E-05**	*−2.42411*
*CLEC5A*	**0.001482**	**3.73E-06**	*−2.21928*	0.072505	0.000688	−1.75105
*COL4A4*	**0.001462**	**3.73E-06**	*−2.18249*	1	0.017007	−1.94555
*PRTG*	**0.00085**	**2.30E-06**	*−2.16704*	**0.000415**	**1.06E-05**	*−3.39011*
*CTSS*	**7.00E-07**	**5.24E-09**	*−2.11030*	1	0.045919	−1.46121
*OSBPL3*	**4.65E-06**	**2.38E-08**	*−2.10227*	0.05406	**0.000583**	−1.67649
*BCL11B*	**0.01734**	**3.27E-05**	*−2.09141*	0.071549	**0.000688**	*−2.19192*
*PRRG1*	**6.71E-07**	**5.16E-09**	*−2.08980*	1	0.025403	−1.67003
*ANTXR2*	**1.94E-05**	**8.44E-08**	*−2.06102*	0.36279	**0.002497**	−1.90464
*PGR*	**0.003108**	**7.26E-06**	*−2.03599*	0.480485	**0.002965**	−1.87897
*BAALC*	**0.005685**	**1.24E-05**	*−2.01197*	**0.009384**	**0.000146**	−1.83602
*LIMD1*	**4.71E-05**	**1.76E-07**	−1.87690	0.617357	**0.003677**	*−2.02599*
*NFIB*	**2.16E-06**	**1.28E-08**	−1.49800	1	0.015881	*−2.01869*

Note: If fold changes in the FF or FFPE sample sets are <-2, they are denoted in italics, respectively. If the Bonferroni P-value or FDR (Q-value) are <0.05 or <0.01, they are labeled in bold font, respectively.

*FF*=Fresh Frozen tissues.

*FFPE*=Formalin-Fixed Paraffin-Embedded tissues.

*FDR*=False Discovery Rate.

**Table 5:** **56 target genes with fold changes**>**2 in either FF or FFPE**

**Gene**	**Bonferroni P-value (FF sample set)**	**FDR (Q-value) (FF sample set)**	**Fold change (FF sample set)**	**Bonferroni P-value (FFPE sample set)**	**FDR(Q-value) (FFPE sample set)**	**Fold change (FFPE sample set)**
*MYT1L*	**5.51E-11**	**3.88E-12**	*12.4550*	**4.53E-13**	**1.16E-13**	*7.39483*
*GRIA2*	**2.45E-09**	**4.06E-11**	*11.9104*	**4.43E-08**	**2.06E-09**	*4.37013*
*GABRB3*	**7.34E-12**	**1.03E-12**	*7.88814*	**1.21E-20**	**6.19E-21**	*7.01235*
*GNG4*	**1.27E-10**	**7.16E-12**	*6.99072*	**0.000262**	**4.07E-06**	*2.79579*
*BSN*	**9.94E-13**	**2.80E-13**	*6.90166*	0.32599	**0.001558**	*1.86761*
*DCX*	**3.25E-07**	**1.99E-09**	*6.89053*	1	**0.004534**	*2.57829*
*ADCY1*	**1.84E-08**	**1.86E-10**	*6.21244*	**0.000107**	**1.96E-06**	*4.32350*
*MAPT*	**1.65E-08**	**1.78E-10**	*5.62091*	**0.006132**	**5.81E-05**	*2.33615*
*ONECUT2*	**6.61E-06**	**2.43E-08**	*5.23250*	**7.35E-11**	**1.25E-11**	*9.43872*
*KIAA1853*	**6.54E-07**	**3.76E-09**	*5.19024*	**0.006683**	**6.04E-05**	*3.04028*
*KIAA2022*	**1.54E-09**	**2.92E-11**	*5.09764*	1	**0.004154**	*2.49768*
*DIO2*	**1.21E-06**	**5.86E-09**	*4.70725*	**4.05E-08**	**2.06E-09**	*4.34805*
*MAP1B*	**5.70E-09**	**7.54E-11**	*4.63902*	**6.45E-05**	**1.32E-06**	*2.70928*
*LMX1B*	**4.11E-06**	**1.61E-08**	*4.52942*	**4.18E-09**	**4.27E-10**	*7.73015*
*GDAP1*	**4.08E-10**	**1.44E-11**	*4.48671*	**4.11E-08**	**2.06E-09**	*4.16645*
*PCSK2*	**2.50E-05**	**7.73E-08**	*3.86742*	**0.009937**	**8.33E-05**	1.89466
*KCNMA1*	**3.23E-11**	**3.03E-12**	*3.67536*	**0.031252**	**0.000228**	1.76140
*NAPB*	0.072657	**8.86E-05**	*3.46819*	0.87771	**0.0034**	*2.01038*
*LONRF2*	**1.99E-10**	**8.00E-12**	*3.44860*	**0.000555**	**7.89E-06**	*2.72746*
*BCL11A*	**6.40E-10**	**1.74E-11**	*3.44466*	**0.006028**	**5.81E-05**	*2.17082*
*NPTXR*	**1.07E-06**	**5.27E-09**	*3.30689*	1	**0.020193**	1.94804
*CNTNAP2*	**0.228461**	**0.000254**	*3.28905*	0.096864	**0.000563**	*3.86958*
*PPM1E*	**0.000311**	**7.63E-07**	*3.27730*	**0.033037**	**0.000231**	*4.02684*
*NRXN1*	**1.05E-07**	**7.43E-10**	*3.26422*	**5.62E-10**	**7.18E-11**	*3.16390*
*HOOK1*	**7.40E-10**	**1.74E-11**	*3.08291*	**3.82E-06**	**1.08E-07**	*2.08999*
*GRIN3A*	**0.002214**	**4.24E-06**	*3.05354*	0.832186	**0.003248**	*2.27896*
*ACVR1C*	**0.000623**	**1.44E-06**	*3.04558*	0.226766	**0.00116**	1.36657
*KCNK10*	**0.001691**	**3.38E-06**	*2.86392*	**0.024849**	**0.000194**	*2.34985*
*GALNT13*	0.063352	**7.97E-05**	*2.64778*	**0.032131**	**0.000228**	*3.08732*
*GRIA3*	**0.005907**	**9.67E-06**	*2.63121*	**0.006349**	**5.90E-05**	*3.00096*
*MARK1*	**2.02E-07**	**1.32E-09**	*2.61530*	0.440426	**0.001905**	*2.17015*
*NMNAT2*	**7.98E-07**	**4.32E-09**	*2.60904*	1	**0.009004**	1.91743
*OPRK1*	**0.034466**	**4.69E-05**	*2.60605*	0.360804	**0.001688**	1.78113
*NBEA*	**0.000203**	**5.16E-07**	*2.54401*	**0.000695**	**9.50E-06**	*2.06122*
*PLEKHA6*	**8.76E-07**	**4.66E-09**	*2.50270*	**0.000165**	**2.78E-06**	*2.74812*
*KIAA0319*	**0.001813**	**3.52E-06**	*2.47998*	0.050027	**0.000319**	*2.93101*
*LASS6*	**2.35E-05**	**7.36E-08**	*2.27989*	0.050576	**0.000319**	*2.59056*
*MRAS*	0.154027	**0.000179**	*2.20056*	1	**0.016486**	1.66357
*LGI2*	**2.03E-05**	**6.48E-08**	*2.19808*	1	0.052341	1.27189
*PCDHAC1*	**0.000116**	**3.05E-07**	*2.14330*	0.157803	**0.000858**	*2.18946*
*NCALD*	**2.54E-06**	**1.10E-08**	*2.08249*	**0.047075**	**0.000305**	1.78080
*PRKACB*	**2.67E-06**	**1.14E-08**	*2.07355*	1	**0.014795**	1.43322
*CDC42BPA*	**1.02E-05**	**3.51E-08**	*2.05556*	**0.008406**	**7.16E-05**	1.85332
*ARNT2*	**0.00032**	**7.78E-07**	*2.05120*	1	**0.008178**	*2.51625*
*ICA1L*	**6.14E-06**	**2.31E-08**	*2.04325*	**0.0004**	**5.85E-06**	*2.04596*
*GREB1*	**0.035592**	**4.82E-05**	1.98638	0.334941	**0.001586**	*2.53911*
*ARL3*	**0.004337**	**7.54E-06**	1.95526	1	**0.008061**	*2.03861*
*RALGPS1*	**3.95E-08**	**3.28E-10**	1.93623	0.136701	**0.000768**	*2.09949*
*AGPAT5*	**0.005877**	**9.67E-06**	1.75658	**0.046976**	**0.000305**	*2.40687*
*CASK*	**0.00097**	**2.11E-06**	1.72623	0.364994	**0.001688**	*2.42452*
*RGS5*	0.598115	**0.000593**	1.57714	0.42732	**0.001868**	*2.13286*
*PHLPPL*	**0.037763**	**5.06E-05**	1.52873	0.66169	**0.002643**	*2.11452*
*KSR2*	**0.007235**	**1.16E-05**	1.52085	**1.90E-08**	**1.62E-09**	*3.17742*
*ADCY2*	**0.000858**	**1.90E-06**	1.38008	**0.00176**	**2.14E-05**	*2.87647*

Note: If fold changes in the FF or FFPE sample sets are <-2, they are denoted in italics, respectively. If the Bonferroni P-value or FDR (Q-value) are <0.05 or <0.01, they are labeled in bold font, respectively.

*FF*=Fresh Frozen tissues.

*FFPE*=Formalin-Fixed Paraffin-Embedded tissues.

*FDR*=False Discovery Rate.

### The expressions of miRNAs and mRNAs in tumors and disease free survival (DFS)

In order to analyze the association between expression levels of miRNAs, mRNA, and clinical outcomes, we combined FF and FFPE together and adjusted sample type as a covariate to calculate the hazard ratio (HR) due to the small numbers of each sample set. Thus, there were a total of 47 cases in the miRNA group and 48 cases in the mRNA group (one patient more in the mRNA group compared to the miRNA group) who had available follow up information. There were five cases with status=1 (death, recurrence or progression). The median follow up time was 6.4 years. All the cases underwent surgery, and one case underwent radiation therapy following surgery. There was no correlation between DFS and clinical-demographical characteristics, i.e., gender, age and stage.

### miRNAs and DFS

We used the expression levels of the miRNAs, which are listed in Table 2 and as the predictors, respectively. However, there was no miRNA with significant predictive power for DFS.

### mRNAs and DFS

We analyzed the predicted mRNAs in tumors, which are listed in Tables 4 and 5. Among the genes with a FC<-2, two genes, i.e., *CREB5* and *PTPRB* had a significant HR, and one gene, *COL4A3*, had a marginally significant HR, suggesting high expressions of these genes remarkably predicted better DFS (Table 6). Additionally, no genes with a FC>2 had significant predictive power for DFS.

**Table 6:** The mRNAs with significant or marginally significant hazard ratios

**mRNAs**	**Fold changes**		**Expressions in tumors and DFS**			
	**FF**	**FFPE**	**HR**	**Low CI**	**Up CI**	**p**-**value**
*CREB5*	−2.29795	−1.80701	0.0327887	0.00147199	0.73037	0.030894^a^
*PTPRB*	−2.54037	−1.76936	0.19048	0.0435739	0.832671	0.0275727^b^
*COL4A3*	−3.29984	−2.31972	0.367349	0.119984	1.1247	0.0794032^c^

Note: Statistical powers of the COX model were calculated by using XLSTAT (Addinsoft Inc., New York, NY, USA) and presented as follows: a. 0.990; b. 0.611; c: 0.635.

*FF*=Fresh Frozen tissues.

*FFPE*=Formalin-Fixed Paraffin-Embedded tissues.

*FDR*=False Discovery Rate.

*DFS*=Disease Free Survival.

*Low CI*=Lower 95% confidence interval.

*UP CI*=Upper 95% Confidence interval.

### Validation of differential expressions of *CREB5*, *PTPRB* and *COL4A3* by using RNA-sequencing (RNA-Seq)

To validate the differential expression of *CREB5*, *PTPRB* and COL4A3, we used RNA-seq to detect the expression of the abovementioned genes in 10 pairs of tumor/normal fresh frozen tissues. Additional file 1: Table S1 shows the Bonferroni-P-values, FDRs (Q-values) and FCs of the abovementioned genes by using DASL (FF sample set) or RNA-seq. All FCs of the mRNAs in DASL can be validated by RNA-seq (Additional file 1: Table S1). We also evaluated the expression data of *CREB5*, *PTPRB* and *COL4A3* from Oncomine [9], indicating that *CREB5*, *PTPRB* and *COL4A3* were significantly lower in carcinoid than normal lung tissues (Additional file 1: Figure S1), which was accordant to the results of the DASL and RNA-seq datasets.

### Gene mutations in PCTs

WES showed 79 mutated genes in 9 carcinoid tumors (Additional file 1: Table S2). *TP53* was the only recurring mutation (2/9). Each of the other genes was only mutated in one case. Intriguingly, some published studies [10-17] (Additional file 1: Table S3) have proved that the mutation of *TP53* may regulate the expression of some top miRNAs listed in Table 3.

## Discussion

The molecular mechanism of the tumorigenesis of PCTs needs to be further investigated. Furthermore, there are very few studies focused on PCTs and miRNAs [18], which hold key roles in the regulation of a variety of cellular processes.

It is well known that dysregulations of miRNAs are important to keep the malignant phenotype of many tumors. The distorted and unique expression profile of miRNAs in different types and subsets of tumors make miRNAs an attractive source of sensitive biomarkers [19]. Importantly, miRNAs not only regulate the expression of oncogenes and tumor suppressors but also play the roles as oncogenes and tumor suppressors. It is very important to screen the expression of these miRNAs in tumor and normal tissues by using miRNA profiling. In our study, we compared the differential expression of 1145 miRNAs between carcinoid tissues vs. matched normal lung tissues, by using miRNA microarray, a high throughput approach. Recently, Kolbert et al. [20] compared the results of miRNA expression levels by using several platforms, including Illumina miRNA arrays and Illumina Next Generation Sequencing, and verified these results with those of quantitative PCR data. It was demonstrated that the within-platform reproducibility for each method was consistently high and detection of miRNA transcripts was similar across multiple platforms. As a result, we believe it is reliable to test the levels of miRNA expression by using Illumnia arrays.

A major shortcoming of gene expression profiling studies on neuroendocrine lung tumors is the absence of a proper reference tissue because pulmonary neuroendocrine cells are only scarcely present in the lung. Therefore, normal adjacent lung tissue is used as a reference.

We obtained the top 16 miRNAs that showed positive FCs and 17 miRNAs with negative FCs, confirmed by either FF or FFPE sample sets. Although we did not identify the top miRNAs with significant predictive power for DFS probably due to the small sample size, we believe that the biological functions of the other top miRNAs in PCTs still warrant further investigations.

By bioinformatics tools, we predicted the target mRNAs, which were derived from the miRNAs with top positive FCs and negative FCs, respectively. Finally, we obtained 44 genes with FC<-2 and 56 genes with FC>2, respectively. The COX proportional hazard model demonstrated that doubled expressions of three genes, i.e., *CREB5*, *PTPRB* and *COL4A3*, was associated with 63% to 97% decreased risk of death, recurrence or progression, suggesting their potential role as tumor suppressors (Table 6). The statistical power of the COX model of three genes were 0.990 (*CREB5*), 0.611 (*PTPRB*) and 0.635 (*COL4A3*), respectively. Therefore, we will confirm the clinical implications of these genes in a large cohort of cases. *PTPRB* has been reported to be a drug addiction-associated gene [21], and *CREB5* to be upregulated in the blood of cases with cluster headaches when compared to controls [22]. High *COL4A3* expression was found to correlate with poor prognosis after combined cisplatin-gemcitabine chemotherapy in non-small cell lung cancer, showing the discrepancy of biological functions of this gene in different tumors [23]. We believe the above mentioned genes, which have remarkable differential expression and significant clinical implications in PCTs, to be potential therapeutic targets. However, the biological functions and clinical utility of these genes warrant further in vitro and in vivo investigations.

Recently, a study of 48 small intestine neuroendocrine tumors by parallel exome sequencing indicated that somatic single nucleotide variants affected a preponderance of cancer genes, including *FGFR2*, *MEN1*, *HOOK3*, *EZH2*, *MLF1*, *CARD11*, *VHL*, *NONO* and *SMAD1*[24]. Our study indicates *TP53* was the only repeated mutation (2/9), and each of the other 78 genes including *HOOK3* were only mutated in one case, probably due to the disparities in different organs. Indeed, the *p53* mutation has been reported in 20% of typical PCTs [25], which is in accordance to our results (2/9 cases). Intriguingly, some published studies [10-17] have proved that the mutation of *p53* may regulate the expression of the miRNAs in a variety of tumors. We postulate that the *p53* mutation may also affect the expression of the miRNAs in PCTs. The underlying associations between the *p53* mutation and miRNAs expression in PCTs warrant further studies.

## Conclusion

We identified that the expressions of three genes have clinical implications in PCTs. The biological functions of these biomarkers warrant further studies.

## Methods

### miRNA expression assay

Four samples were excluded due to low quality. A group of 47 carcinoids tumors were analyzed; each with paired tumor-adjacent normal tissue samples (at least 5 cm away from the primary tumors). There are 24 pairs of tumor/normal samples in FF tissues, and 23 pairs of tumor/normal samples in FFPE. The clinical and demographical features of these cases are shown in Table 1. The expression status of 1145 miRNAs was generated by Illumina miRNA Arrays (San Diego, CA, U.S.A.), as described before [20]. We deposited the miRNA profiling data to Gene Expression Omnibus (GEO, accession number: GSE58600).

To explore the technical and biological factors that may affect miRNA expression levels, we performed multiple factor ANOVA analysis to identify SOV. To visualize the miRNA expression in tumor and normal tissues, we projected the numeric data by using scatter plot, i.e., PCA.

### mRNA expression assay

A total of 51 carcinoid tumors were analyzed by the whole-genome DASL Assay (Illumina Beadchips Array, San Diego, CA, U.S.A.) as described before [26], each with paired tumor-adjacent normal tissue. There are 25 pairs of tumor/normal in FF tissues, and 26 pairs of tumor/normal samples in FFPE tissues. Additionally, nine pairs of tumor/normal samples in FF tissues were analyzed by whole transcriptome sequencing (RNA-seq) using the TruSeq protocol (Illumina, San Diego, CA, U.S.A.). The clinical and demographical features of these cases are shown in Table 1. Finally, the differential expressions of mRNAs were validated by Oncomine (a public cancer microarray database; https://www.oncomine.org/).

### Prediction of target mRNA of miRNA

We obtained the target mRNAs of the miRNA using the bioinformatics prediction tool, “miRWalk” (http://www.umm.uni-heidelberg.de/apps/zmf/mirwalk/) [27]. miRWalk’s website also provided other prediction tools for crosschecking. These tools include, miRanda [28], miRDB [29], RNA22 [30] and Targetscan [31].

### Whole exome sequencing (WES) for gene mutation identification

Genomic DNA was purified from 9 pairs of carcinoid tumors and matched normal tissues. The nine cases were selected from the 51 cases that were analyzed for mRNA expression profiling. The clinical and demographical features of these cases are shown in Table 1. DNA samples were enriched for coding regions in the genome using custom DNA capture (SureSelect, Agilent Technologies, Santa Clara, CA, U.S.A.) and sequenced using HiSeq2000 (Illumina, San Diego, CA, U.S.A.) at an average depth of 1017×. Mutation status in the samples were analyzed by algorithms developed by Personal Genome Diagnostics Inc. (Baltimore, MD, U.S.A.). WES data were mapped to the reference human genome sequence and sequence alterations were determined by comparison of over 50 million bases of tumor and normal DNA.

### Survival analysis

Follow-up was conducted through detailed medical records data abstraction and self-administered questionnaires, starting within six months post diagnosis and then annually, thereafter. For deceased patients, the follow-up questionnaire was sent to the next-of-kin to acquire the death information, e.g., death date. We used disease free survival (DFS) as progression-free or recurrence-free survival from the operation. Recurrence, progression or death were counted as events. We evaluated the statistical power for survival analysis, considering numbers of events and sample size and Hazard ratio (HR).

### Data analysis

The statistical analyses were performed using the analysis of variance (ANOVA) to analyze differential expression of miRNA and mRNA between tumors and normal tissues. Fold changes (FCs) of miRNA and mRNA differential expression were examined between tumors vs. normal tissues using stringent thresholds controlling for false discovery rates. Hence, a positive FC meant the expression level in tumor is higher than in normal, and a negative FC meant the expression level in the tumor is lower than in normal. Thereafter, multiple test corrections of the P value, i.e., Bonferroni P-value and FDR-Q-value, were analyzed. All of the aforesaid calculations were performed using SPSS Version 20.0 software for Windows (IBM Inc., New York, NY, U.S.A.) and Partek Genomics Suite 6.6 software (Partek Inc., St. Louis, MO, U.S.A.). Additionally, prognostic factors were examined by univariate and multivariate analyses using a Cox proportional hazards model, and the statistical powers were evaluated by XLSTAT (Addinsoft Inc., New York, U.S.A.). A value of p<0.05 (two-sided) was considered statistically significant.

## Competing interests

The authors have declared that they have no competing interests.

## Authors’ contributions

BD carried out the data analysis and drafted the manuscript. JM carried out the data analysis and revised the manuscript. MCA carried out the data analysis, reviewed the slide, and revised the manuscript. ZS carried out the data analysis and revised the manuscript. LW carried out the genetic studies and revised the manuscript. BE carried out the data analysis and revised the manuscript. MY carried out the data analysis and revised the manuscript. EW carried out the data analysis and revised the manuscript. JJ carried out the genetic studies and revised the manuscript. DW collected the samples. PY designed the study, guided the analyses and results interpretation, and revised the manuscript. All authors read and approved the final manuscript.

## Supplementary Material

Additional file 1: Table S1 Validation of CREB5, PTPRB and COL4A3 by using DASL assay and RNA-sequencing. **Table S2.** Gene mutations in 9 pulmonary carcinoids detected by Exon-Seq. **Table S3.** The published results of the correlation between p53 mutation or function and miRNA expression. **Figure S1.** SOV of miRNA expression and PCA plot of miRNA data in FF and FFPE sample sets. **Figure S2.** mRNA expressions of CREB5, PTPRB and COL4A3 in carcinoid tumors and matched normal tissues in Oncomine dataset. Click here for file
