# Supplementary material for: Clinical biomarkers of pulmonary carcinoid tumors in never smokers via profiling miRNA and target mRNA
Source: Cell Biosci. 2014 Jul 9;4:35. doi: 10.1186/2045-3701-4-35 (PMC4124500; doi:10.1186/2045-3701-4-35)
Supplement: Additional file 1: Table S1 — Validation of CREB5, PTPRB and COL4A3 by using DASL assay and RNA-sequencing. Table S2. Gene mutations in 9 pulmonary carcinoids detected by Exon-Seq. Table S3. The published results of the correlation between p53 mutation or function and miRNA expression. Figure S1. SOV of miRNA expression and PCA plot of miRNA data in FF and FFPE sample sets. Figure S2. mRNA expressions of CREB5, PTPRB and COL4A3 in carcinoid tumors and matched normal tissues in Oncomine dataset. [file 2045-3701-4-35-S1.doc]

**Supplemental Table 1. Validation of CREB5, PTPRB and COL4A3 by using DASL assay and RNA-sequencing**

| **Genes** | **Bonferroni P-value**  **(DASL)** | **FDR(Q-value)**  **(DASL)** | **Fold Change**  **(DASL)** | **Bonferroni P-value**  **(RNA-seq)** | **FDR(Q-value)**  **(RNA-seq)** | **Fold Change**  **(RNA-seq)** |
| --- | --- | --- | --- | --- | --- | --- |
| *CREB5* | 7.68E-09 | 1.40E-10 | -2.29795 | 5.33E-05 | 9.80E-06 | -2.61144 |
| *PTPRB* | 2.98E-07 | 2.86E-09 | -2.54037 | 7.51E-06 | 1.38E-06 | -5.17037 |
| *COL4A3* | 2.88E-06 | 1.62E-08 | -3.29984 | 0.000109 | 2.00E-05 | -3.86125 |

**Supplemental Table 2. Gene mutations in 9 pulmonary carcinoids detected by Exon-Seq.**

| **Gene Symbol** | **Amino Acid (protein)** | **Mutation Type** | **Consequence** | **Case(s) with mutation** |
| --- | --- | --- | --- | --- |
| *TP53* | 181R>S | Substitution | Nonsynonymous coding | 2 |
|  | 156R>S | Substitution | Nonsynonymous coding |  |
| *CCNF* | 170E>K | Substitution | Nonsynonymous coding | 1 |
| *CPNE4* | 240D>N | Substitution | Nonsynonymous coding | 1 |
| *CSN1S1* | 89S> | Deletion | In-frame deletion | 1 |
| *CXCR5* | NA | Insertion | Frameshift | 1 |
| *FRAS1* | 458F>I | Substitution | Nonsynonymous coding | 1 |
| *HOOK3* | NA | Deletion | Frameshift | 1 |
| *IGKV4-1* | 98T>S | Substitution | Nonsynonymous coding | 1 |
| *KIAA1244* | 1074S>N | Substitution | Nonsynonymous coding | 1 |
| *KLK13* | 263R>X | Substitution | Nonsense | 1 |
| *KRT85* | 54R>C | Substitution | Nonsynonymous coding | 1 |
| *MAP7* | 138R>C | Substitution | Nonsynonymous coding | 1 |
| *NFIB* | 127V>I | Substitution | Nonsynonymous coding | 1 |
| *PCLO* | 863S>C | Substitution | Nonsynonymous coding | 1 |
| *RIMKLB* | 267M>I | Substitution | Nonsynonymous coding | 1 |
| *SEMA5B* | 898E>A | Substitution | Nonsynonymous coding | 1 |
| *SLC6A5* | 392I>F | Substitution | Nonsynonymous coding | 1 |
| *SRRM2* | 217K>Q | Substitution | Nonsynonymous coding | 1 |
| *TAAR5* | 313R>W | Substitution | Nonsynonymous coding | 1 |
| *TMEM213* | 107A>V | Substitution | Nonsynonymous coding | 1 |
| *TNFAIP3* | 415Q>X | Substitution | Nonsense | 1 |
| *TRPV5* | 230Q>K | Substitution | Nonsynonymous coding | 1 |
| *VAV2* | 205E>K | Substitution | Nonsynonymous coding | 1 |
| *ABCA9* | 1397A>T | Substitution | Nonsynonymous coding | 1 |
| *ARID1A* | NA | Insertion | Frameshift | 1 |
| *DNAJC11* | NA | Deletion | Frameshift | 1 |
| *F8* | 208T>A | Substitution | Nonsynonymous coding | 1 |
| *FBN2* | 2124A>G | Substitution | Nonsynonymous coding | 1 |
| *GALNT10* | 460W>X | Substitution | Nonsense | 1 |
| *HSFX1* | 165R>W | Substitution | Nonsynonymous coding | 1 |
| *HSFX1* | 165R>W | Substitution | Nonsynonymous coding | 1 |
| *MBD6* | 844P>R | Substitution | Nonsynonymous coding | 1 |
| *PTHLH* | NA | Substitution | Splice site donor | 1 |
| *RANBP2* | 1809P>R | Substitution | Nonsynonymous coding | 1 |
| *RANBP2* | NA | Deletion | Frameshift | 1 |
| *ZBTB33* | 215A>V | Substitution | Nonsynonymous coding | 1 |
| *ZNF74* | 130KE>K | Deletion | In-frame deletion | 1 |
| *ABI3BP* | NA | Insertion | Splice site acceptor | 1 |
| *AC007731.16* | 240T>M | Substitution | Nonsynonymous coding | 1 |
| *AC091435.3* | 245A>V | Substitution | Nonsynonymous coding | 1 |
| *BRIP1* | 1018K>T | Substitution | Nonsynonymous coding | 1 |
| *C21orf41* | 35G>R | Substitution | Nonsynonymous coding | 1 |
| *CSRNP3* | 222P>L | Substitution | Nonsynonymous coding | 1 |
| *ECD* | NA | Deletion | Splice site acceptor | 1 |
| *FXR1* | 275A>P | Substitution | Nonsynonymous coding | 1 |
| *SUPT6H* | 17N>D | Substitution | Nonsynonymous coding | 1 |
| *TKTL1* | 110G>D | Substitution | Nonsynonymous coding | 1 |
| *BACE2* | 516R>H | Substitution | Nonsynonymous coding | 1 |
| *CENPT* | 269A>D | Substitution | Nonsynonymous coding | 1 |
| *CHTF8* | 347H>N | Substitution | Nonsynonymous coding | 1 |
| *ERBB2* | 848A>D | Substitution | Nonsynonymous coding | 1 |
| *FIG4* | 107I>S | Substitution | Nonsynonymous coding | 1 |
| *GPR119* | 71R>W | Substitution | Nonsynonymous coding | 1 |
| *SAMD11* | NA | Insertion | Frameshift | 1 |
| *ZNF689* | 346A>S | Substitution | Nonsynonymous coding | 1 |
| *AGFG1* | NA | Substitution | Splice site acceptor | 1 |
| *MYO1F* | 652G>S | Substitution | Nonsynonymous coding | 1 |
| *RUFY1* | 484I>V | Substitution | Nonsynonymous coding | 1 |
| *AHSG* | 267A>T | Substitution | Nonsynonymous coding | 1 |
| *ZDBF2* | 1077D>N | Substitution | Nonsynonymous coding | 1 |
| *C14orf104* | 298A>T | Substitution | Nonsynonymous coding | 1 |
| *C2orf53* | 90S>C | Substitution | Nonsynonymous coding | 1 |
| *CCNB3* | 765Q>K | Substitution | Nonsynonymous coding | 1 |
| *DHX15* | 402E>D | Substitution | Nonsynonymous coding | 1 |
| *DNAH17* | 1888G>V | Substitution | Nonsynonymous coding | 1 |
| *DOPEY2* | 540Q>X | Substitution | Nonsense | 1 |
| *EXOC3* | 150C>Y | Substitution | Nonsynonymous coding | 1 |
| *HERC1* | 1043N>S | Substitution | Nonsynonymous coding | 1 |
| *PTPRR* | 106V>E | Substitution | Nonsynonymous coding | 1 |
| *TRIM55* | 210E>D | Substitution | Nonsynonymous coding | 1 |
| *WDFY3* | 1908R>L | Substitution | Nonsynonymous coding | 1 |
| *WDR19* | NA | Substitution | Splice site donor | 1 |
| *ZC3H18* | NA | Deletion | Splice site donor | 1 |
| *CLIP1* | 281I>T | Substitution | Nonsynonymous coding | 1 |
| *EGLN3* | 123A>S | Substitution | Nonsynonymous coding | 1 |
| *NUP153* | NA | Deletion | Splice site acceptor | 1 |
| *RYR1* | 280R>Q | Substitution | Nonsynonymous coding | 1 |
| *SEL1L3* | NA | Deletion | Splice site acceptor | 1 |
| *SPTA1* | 1493R>Q | Substitution | Nonsynonymous coding | 1 |
| *RASGRP2* | 380L>M | Substitution | Nonsynonymous coding | 1 |
| *TLR6* | 377V>A | Substitution | Nonsynonymous coding | 1 |

**Supplemental Table 3. The published results of the correlation between p53 mutation or function and miRNA expression**

| **miRNA** | **Fold Change**  **in pulmonary carcinoid** | **Correlation between Tp53 mutation or function and miRNA expression** |
| --- | --- | --- |
| miR-203 | **FC<0** | miR-203 expression was reduced where p53 function was compromised in human foreskin keratinocytes. [17] |
| miR-155 | **FC<0** | Mutated p53 drove invasion in breast tumors through up-regulation of miR-155. [18] |
| miR-34b | **FC<0** | miR-34b is target of p53, and it was down-regulated in p53-null human ovarian carcinoma cells.[19] |
| miR-181b | **FC<0** | Sequencing analysis revealed that miR-181b expression was strongly associated with the mutation status of the p53 gene in colorectal cancer. [20] |
| miR-34b* | **FC<0** | The miR-34 family is directly transactivated by P53, which is frequently mutated in human epithelial ovarian cancer. [21] |
| miR-34c-3p | **FC<0** | A significant inhibition was observed only for miR-34c-3p, as an effector of P53, on SiHa cells migration and invasion. [22] |
| miR-146a | **FC<0** | Elevated p53 decreases the expression of miR-146a, while knocking down p53 increases miR-146a expressions in STHdh(Q7)/Hdh(Q7) cells, i.e., cells model of Huntington's disease. [23] |
| miR-1 | **FC<0** | mir-1 showed statistically significant alterations in expression levels in p53 -/- embryos compared to p53 +/+ embryos. [24] |

**
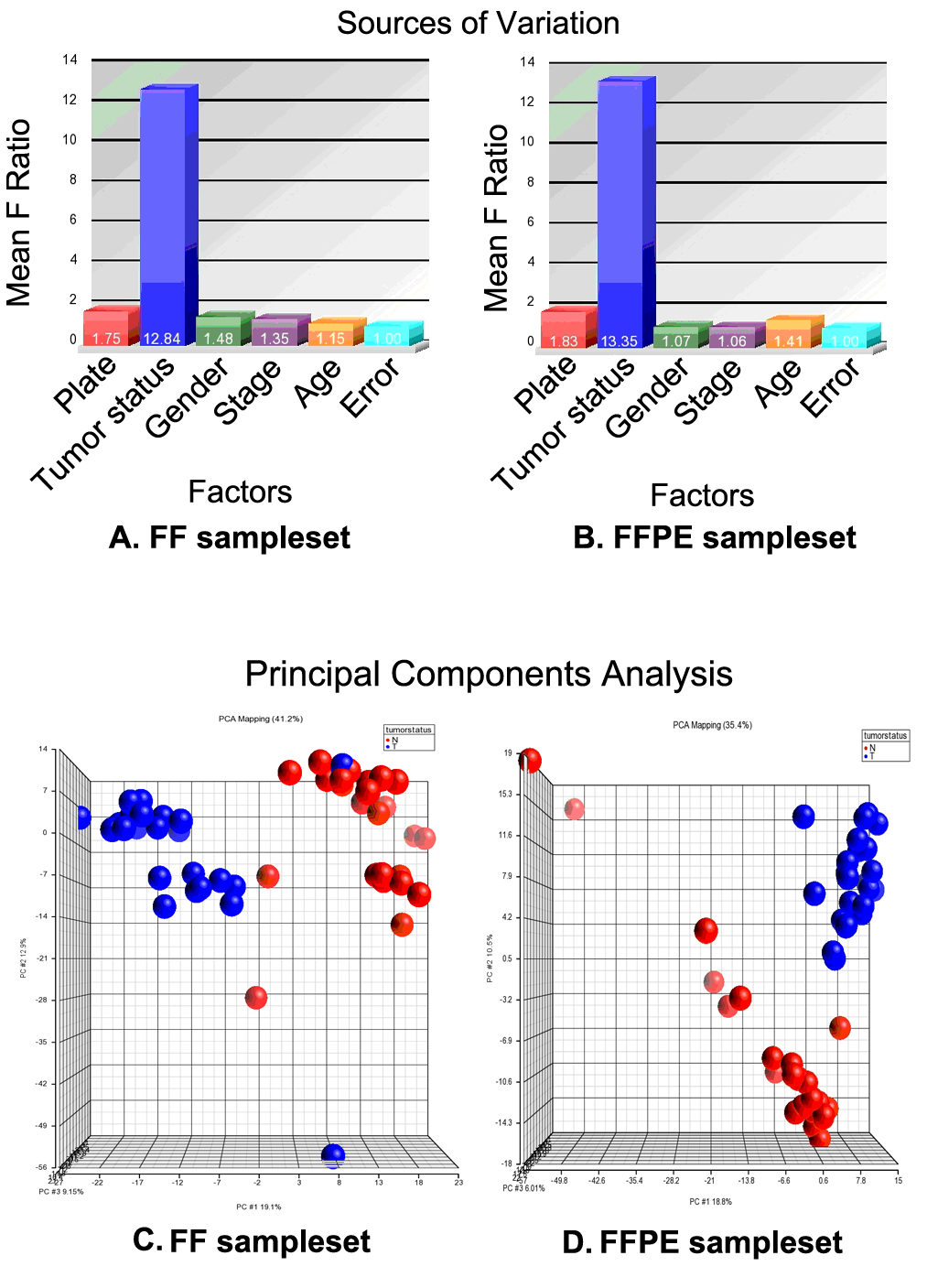
**

**Supplemental Figure 1. SOV of miRNA expression and PCA plot of miRNA data in FF and FFPE sample sets.**

**Note:** Figure1 A and B show sources of variation of miRNAs expression in FF and FFPE sample sets, respectively. Figure 1 C and D show principal components analysis of miRNAs expression in FF and FFPE sample sets respectively. In Figure 1 C and D, blue and red dots represent tumors and normal tissues, respectively. Each dot represents a sample with merged miRNAs.

FF=Fresh Frozen tissues; FFPE=Formalin-Fixed Paraffin-Embedded tissues

**
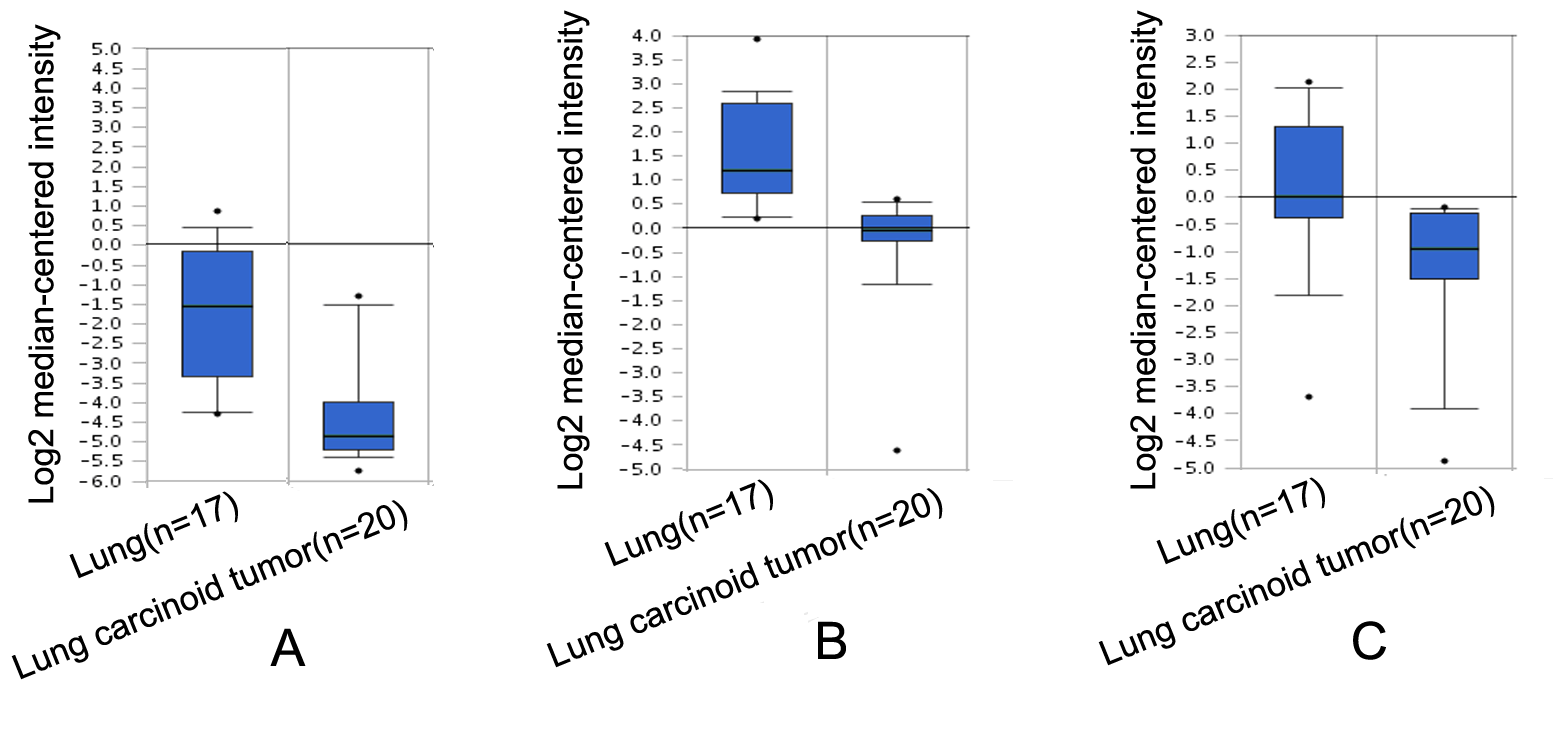
**

**Supplemental Figure 2. mRNA expressions of CREB5, PTPRB and COL4A3 in carcinoid tumors and matched normal tissues in Oncomine dataset.**

**Note:** A: CREB5; B: PTPRB; C: COL4A3

**Reference**

17. McKenna DJ, McDade SS, Patel D, McCance DJ: **MicroRNA 203 expression in keratinocytes is dependent on regulation of p53 levels by E6.** *J Virol* 2010, **84:**10644-10652.

18. Neilsen PM, Noll JE, Mattiske S, Bracken CP, Gregory PA, Schulz RB, Lim SP, Kumar R, Suetani RJ, Goodall GJ, Callen DF: **Mutant p53 drives invasion in breast tumors through up-regulation of miR-155.** *Oncogene* 2013, **32:**2992-3000.

19. Corney DC, Flesken-Nikitin A, Godwin AK, Wang W, Nikitin AY: **MicroRNA-34b and MicroRNA-34c are targets of p53 and cooperate in control of cell proliferation and adhesion-independent growth.** *Cancer Res* 2007, **67:**8433-8438.

20. Xi Y, Formentini A, Chien M, Weir DB, Russo JJ, Ju J, Kornmann M: **Prognostic Values of microRNAs in Colorectal Cancer.** *Biomark Insights* 2006, **2:**113-121.

21. Corney DC, Hwang CI, Matoso A, Vogt M, Flesken-Nikitin A, Godwin AK, Kamat AA, Sood AK, Ellenson LH, Hermeking H, Nikitin AY: **Frequent downregulation of miR-34 family in human ovarian cancers.** *Clin Cancer Res* 2010, **16:**1119-1128.

22. Lopez JA, Alvarez-Salas LM: **Differential effects of miR-34c-3p and miR-34c-5p on SiHa cells proliferation apoptosis, migration and invasion.** *Biochem Biophys Res Commun* 2011, **409:**513-519.

23. Ghose J, Sinha M, Das E, Jana NR, Bhattacharyya NP: **Regulation of miR-146a by RelA/NFkB and p53 in STHdh(Q111)/Hdh(Q111) cells, a cell model of Huntington's disease.** *PLoS One* 2011, **6:**e23837.

24. Hosako H, Martin GS, Barrier M, Chen YA, Ivanov IV, Mirkes PE: **Gene and microRNA expression in p53-deficient day 8.5 mouse embryos.** *Birth Defects Res A Clin Mol Teratol* 2009, **85:**546-555.
